# Supplementary material for: Macromolecule Translocation across the Intestinal Mucosa of HIV-Infected Patients by Transcytosis and through Apoptotic Leaks
Source: Cells. 2023 Jul 18;12(14):1887. doi: 10.3390/cells12141887 (PMC10378197; doi:10.3390/cells12141887)
Supplement: Supplementary file 1 [file cells-12-01887-s001.zip › cells-2481940-supplementary.pdf]

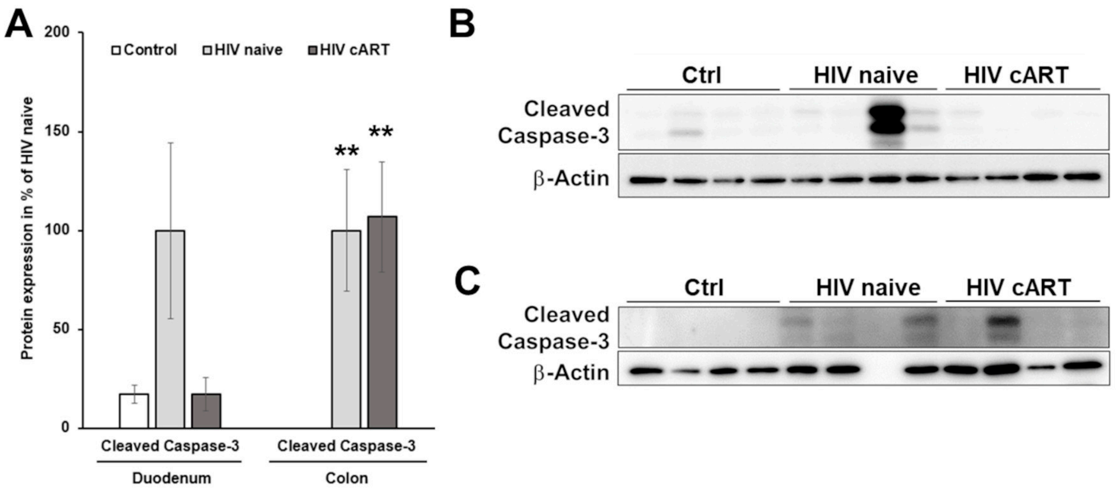


Figure S1: apoptosis in the duodenal and colon mucosa of HIV-infected patients and HIV-negative controls quantified as an expression of cleaved Caspase-3.
